# Human Autopsy-Derived Scalp Fibroblast Biobanking for Age-Related Neurodegenerative Disease Research

**DOI:** 10.3390/cells9112383

**Published:** 2020-10-30

**Authors:** Suet Theng Beh, Carlye Frisch, David A. Brafman, Jared Churko, Jessica E. Walker, Geidy E. Serrano, Lucia I. Sue, Eric M. Reiman, Thomas G. Beach, Lih-Fen Lue

**Affiliations:** 1 Banner Sun Health Research Institute, Sun City, AZ 85351, USA; SuetTheng.Beh@bannerhealth.com (S.T.B.); Jessica.Walker@bannerhealth.com (J.E.W.); Geidy.Serrano@bannerhealth.com (G.E.S.); Lucia.Sue@bannerhealth.com (L.I.S.); Thomas.Beach@bannerhealth.com (T.G.B.); 2 School of Biological and Health Systems Engineering, Arizona State University, 501 E. Tyler Mall, ECG 334A, Tempe, AZ 85287, USA; cafrisch@asu.edu (C.F.); David.Brafman@asu.edu (D.A.B.); 3 Department of Cellular and Molecular Medicine, University of Arizona, Tucson, AZ 85721, USA; jchurko@email.arizona.edu; 4 Banner Alzheimer’s Institute, Phoenix, AZ 85006, USA; Eric.Reiman@bannerhealth.com

**Keywords:** Alzheimer’s disease, Parkinson’s disease, postmortem, scalp explant, fibroblasts, apolipoprotein E genotype, human inducible pluripotent stem cells, C9orf72

## Abstract

The Arizona Study of Aging and Neurodegenerative Disorders/Brain and Body Donation Program at Banner Sun Health Research Institute (BSHRI) is a longitudinal clinicopathological study with a current enrollment of more than 900 living subjects for aging and neurodegenerative disease research. Annual clinical assessments are done by cognitive and movement neurologists and neuropsychologists. Brain and body tissues are collected at a median postmortem interval of 3.0 h for neuropathological diagnosis and banking. Since 2018, the program has undertaken banking of scalp fibroblasts derived from neuropathologically characterized donors with Alzheimer’s disease, Parkinson’s disease, and other neurodegenerative diseases. Here, we describe the procedure development and cell characteristics from 14 male and 15 female donors (mean ± SD of age: 83.6 ± 12.2). Fibroblasts from explant cultures were banked at passage 3. The results of mRNA analysis showed positive expression of fibroblast activation protein, vimentin, fibronectin, and THY1 cell surface antigen. We also demonstrated that the banked fibroblasts from a postmortem elderly donor were successfully reprogramed to human-induced pluripotent stem cells (hiPSCs). Taken together, we have demonstrated the successful establishment of a human autopsy-derived fibroblast banking program. The cryogenically preserved cells are available for request at the program website of the BSHRI.

## 1. Introduction

Sporadic Alzheimer’s disease (AD) and Parkinson’s disease (PD) are the two major neurodegenerative diseases in the aging population. Although much progress has been made in understanding disease mechanisms and identifying therapeutic targets in the last three decades, there are still no effective disease-modifying treatments. Discussions on the lack of translation from animal studies to humans have brought up complex issues of disease modeling [1,2,3,4]. In addition to an incomplete understanding of pathogenic mechanisms and their relationship in human conditions, there are also issues from inherent differences at the levels of genome, function, development, and aging between rodents and humans.

To address species gaps, human cells generated from stem cell technology have been increasingly used. A multitude of publications have reported success in the generation of human-induced pluripotent stem cells (hiPSCs) and subsequent differentiation of neural cells [5,6,7]. Fibroblasts have also been successfully converted to neurons and neural stem cells through direct reprogramming techniques [8,9,10,11]. In addition, human skin fibroblast cultures have been frequently used to study disease mechanisms [12,13,14], biomarkers [15,16,17,18], and therapeutics [19,20].

To provide skin fibroblasts for research purposes, the Brain and Body Donation Program (BBDP) of Banner Sun Health Research Institute (BSHRI) has added a Human Cells Core in 2018, aiming to bank postmortem scalp-derived fibroblasts from clinically and neuropathologically characterized donors. BSHRI has a well-established longitudinal clinicopathological study, the Arizona Study of Aging and Neurodegenerative Disease and BBDPthat banks each year human brain tissues from 60–80 autopsy cases [21]. The participants of the program receive a battery of clinical and neuropsychological assessments during annual visits at BSHRI. After the death of the participants, brain and body tissues are collected for banking and neuropathological characterization including the diagnoses of AD, PD, and other neurodegenerative diseases. The program has served researchers in the academic and pharmaceutical industry by providing neuropathologically-confirmed tissues and biofluids in the last three decades (https://www.brainandbodydonationregistration.org/).

In this report, we described the procedure developed to obtain fibroblasts routinely from postmortem scalp tissues and the characteristics of the fibroblasts banked from the first 29 cases. We also demonstrated the generation of hiPSCs from the cryogenic fibroblasts derived from a 90-year old case.

## 2. Materials and Methods

### 2.1. Ethical Statement

Human scalp samples were collected at autopsy according to the Western Institutional Research Board approved protocol (WIRB^®^ Protocol #20120821) to the Brain and Body Donation Program of Banner Sun Health Research Institute.

### 2.2. Clinical and Neuropathological Diagnosis

The procedures of annual clinical and neuropsychological assessments and autopsy were described in our previous publication [21,22]. Neuropathological diagnosis was based on international consensus criteria including the National Institute on Aging and the Alzheimer’s Association (NIA-AA) criteria for normal controls (NC) and AD. The diagnosis criteria for dementia with Lewy bodies (DLB) and PD were based on a previous publication [23]. The Unified Staging System for Lewy Body (LB) disorders was used to classify the stages of α-synucleinopathy [21]. The densities of amyloid plaques and neurofibrillary tangles were assessed on 40-µm-thick, 10% neural-buffered formalin-fixed brain tissues that were processed for Gallyas, Campbell-Switzer, and thioflavin S stains. The AD pathology was evaluated by a scale of 0–3 based on 0 being no plaques or tangles, 1 being few plaques or tangles, 2 being moderate numbers of plaques and tangles, and 3 being numerous plaques and tangles. The sum of the scores across the five brain regions (frontal, parietal, temporal, and entorhinal cortices and hippocampus) gave 0–15 total scores for plaques and tangles respectively. For LB staging, immunohistochemical staining of phosphorylated α-Synuclein (p-αSyn) was performed on paraffin-embedded sections. The density of LB pathology was assessed in 10 brain regions based on our published system that rates LB pathology on a scale of 0–4 [24]. Examples of the histopathological density of AD (amyloid plaques and neurofibrillary tangles) and LB are shown in Supplemental Figures S1–S3.

### 2.3. Features of the Donors

During 2018–2020, we established the procedure to culture fibroblasts from postmortem scalp tissues. The cells have been banked from 29 donors: 6 in 2018, 14 in 2019, and 9 cases in the first four months of 2020. A summary of age, gender, APOE genotype, years of neurological diagnosis, and age and years of dementia from these cases is shown in Table 1, while the clinical and neuropathological features are summarized in Table 2. These features for the individual donor are detailed in Supplemental Table S1.

### 2.4. Tissue Processing and Culture

**Figure 1:**
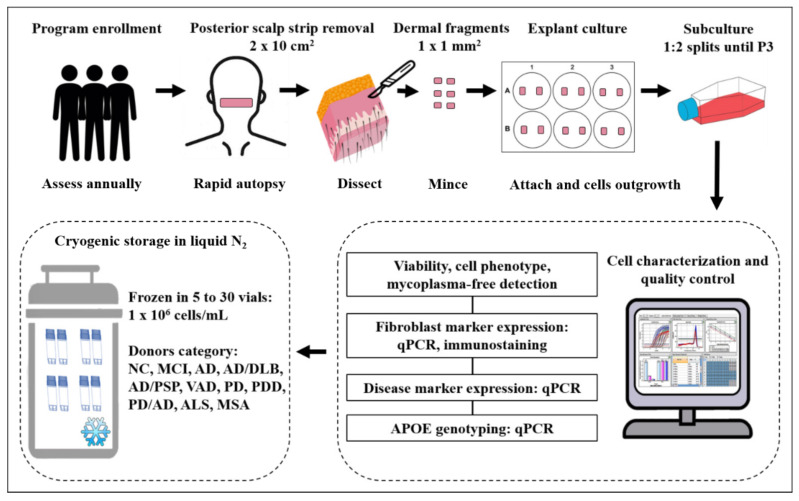
The workflow established in this study for postmortem human fibroblast banking at the Brain and Body Donation Program of Banner Sun Health Research Institute.

The tissue removal for the scalp-derived fibroblast banking program was handled by a 24-h on-call autopsy team. The processing of scalp tissues and culture procedure were performed by the cell culture team in a cell culture facility on the BSHRI campus. The workflow is shown in Figure 1.

At autopsy, posterior scalp samples were dissected before the skull was opened for brain removal. The median postmortem interval (PMI) (hours of delay from death to autopsy) and tissue processing interval (TPI) (hours of delay from death to scalp processing) from 29 donors were 3.8 h and 13.5 h respectively.

The scalp tissue sample was obtained from the posterior side of the head. Immediately after removal, scalp tissues sized 2 × 10 cm^2^, were immersed in a 50-mL plastic tube pre-filled with Hibernate-A solution containing gentamicin and antimycotics (Thermo Fisher Scientific, Waltham, MA, USA). All containers were labeled mentioning time/date, size and weight of the sample, and autopsy number. The containers were stored at 4 °C until ready to process. For tissues that are not processed within the next few days, we used a home-made cryoprotectant to store tissues at −20 °C. The home-made cryoprotectant consisted of 15% Hibernate-A, 0.5X B27 (Thermo Fisher Scientific) or N21-Max media supplement (R and D Systems, Minneapolis, MN, USA), 50% fetal bovine serum (VWR International, Visalia, CA, USA), 0.2 M of Trehalose solution (Life Sciences Advanced Technologies, St Petersburg, FL, USA), and 10% DMSO (Sigma–Aldrich, St. Louis, MO, USA). At the time of processing, scalp tissues were taken out of storage solution and washed three times with cold Wash Buffer [Dulbecco’s Modified Eagle Medium (DMEM) with high glucose (Thermo Fisher Scientific) containing 50 µg/mL gentamicin and 1× antibiotics-antimycotics solution (Thermo Fisher Scientific)]. Hairs were trimmed from the surface of epidermal layer, followed by the removal of adipose tissues ventral to the dermal layer.

Epidermal and dermal tissues were then rinsed with Wash Buffer and placed in a new petri dish. Tissues were cut through both layers into 0.5–1 × 5 cm^2^ strips. The strips were placed inside a petri dish with epidermal layer down and dermal layer up. Using a sharp razor blade, strips of tissue were then cut in a crosshatched pattern into the dermal layer without cutting through the epidermal layer, and then removed from the epidermal layer and kept moist in a small amount of Wash Buffer till completion of dissection. The sizes of tissue pieces were smaller than 1 mm^2^. They were resuspended in Growth Media, FibroLife S2 Growth Media supplemented with LifeFactors (Lifeline Cell Technologies, Frederick, MD, USA) containing recombinant human FGF-basic, recombinant human insulin, L-glutamine, hydrocortisone hemi-succinate, ascorbic acid, fetal bovine serum, gentamicin, and amphotericin B. The detailed formulation of the ingredients is available on the specification sheet (Cat# LL-0011).

Centrifugation was performed to collect the dermal tissues. After removing the liquid, tissues were placed in a petri dish. Two pieces of dermal tissues were transferred to each well of 6-well tissue-culture treated polystyrene plates (Greiner Bio-One North America Inc., Monroe, NC, USA), immersed in approximately 50 µL of Growth Media, and incubated at 37 °C in a humidified atmosphere of 5% CO_2_. Within four hours, more media were added dropwise to individual explant tissues, but not to flood the well. The next day, 1 mL of Growth Media was added to the wells. For maintenance, fresh Growth Media were added once a week until the explants were firmly attached. For the explants that remained unattached, half of the spent media was exchanged with fresh media once a week until attachment or disposal of the explants. Typically, scalp explants were disposed if they did not attach after 6 weeks. Cells were fed with fresh Growth Media twice a week. The changes in explant appearance and cell outgrowth were observed routinely with a phase-contrast light microscope and the results chronologically documented.

### 2.5. Passage and Cryoprotection

When cells reached 80% confluency in the original wells containing explants, they were detached by treating with a trypsin-like recombinant protease called TrypLE Express enzyme (Thermo Fisher Scientific) at 37 °C for 5 min in an incubator. The TrypLE Express enzyme is a ready-to-use proteolytic solution, containing 1 mM EDTA. Detached cells were collected into Growth Media and replated in T75 flasks in a growth area ratio of 1:2. These were the first passage cells (P1) from the explants. When P1 cells were proliferated to 80–85% confluence, they were detached and plated into new flasks to expand at the same ratio for P2. The same procedure was carried out until the P3 culture.

Confluent P3 cells were detached, counted, and aliquoted into 2-mL microcentrifuge tubes. An aliquot of 0.1 million cells was used for plating to chamber slides for morphological characterization. Three aliquots of 0.3 million cells were centrifuged to pellet for RNA, DNA, or protein extraction. For cryoprotection, aliquots of 0.5 × 10^6^–1.0 × 10^6^ cells were centrifuged and resuspended at 1 × 10^6^ cells/mL in commercially formulated cryoprotectant, CryoStor CS5 (Stemcell Technologies, Vancouver, BC, Canada) or FrostaLife Cryopreservation Solution (Lifeline Cell Technology).

Cryoprotected cell aliquots were stored at −80°C for 18–24 h in Mr. Frosty freezing containers filled with isopropanol before transferring to a liquid nitrogen tank for long-term storage in vapor phase. Liquid nitrogen level and interior temperature of the tank were monitored by an alarm system (Primex Inc, Lake Geneva, WI, USA). Cell pellets for characterization purposes were stored at −80 °C until analysis. All banked cells were serially documented from the time of autopsy to cryogenic storage. The information documented included the initial number of explant wells, the date of first observation of cell outgrowth, expansion, cell yield, number of cryogenic vials, and storage location in cryogenic boxes in liquid nitrogen tank.

### 2.6. Recovery Rate of Cryoprotected Fibroblasts

To determine the recovery rate, periodically, cryoprotected fibroblasts were thawed. Cells were immediately counted with a hemocytometer using a trypan blue dye exclusion method. Cells that excluded the dye were counted as live cells. The percentages of live cells in the total cell numbers were obtained. Cells recovered from the thawing procedure were assessed for attachment and growth within 24 h.

### 2.7. Immunohistochemistry, Immunofluorescence, and Microscopy

The details of the brain dissection and diagnostic stains for morphological characterization were described in our previous publication [21]. Briefly, sections of 6 μm were taken on a rotary microtome and immunohistochemically stained for p-αSyn for Lewy bodies and Lewy-related neurites. Pre-cut free-floating 40 μm sections were specific stained with Campbell-Switzer and Gallyas methods for amyloid plaques and neurofibrillary tangles, respectively. Stained tissue sections were examined and photographed using a Leica DM2500 microscope. For morphological characterization, cells at P3 were cultured in 8-chamber slides (Falcon through VWR International) at 1000 cells per chamber. The attached cells were rinsed with pH 7.4 phosphate-buffered saline (PBS) before fixing with 4% paraformaldehyde in PBS for 15 min at room temperature. Before immunolabeling, cells were washed twice in 0.1% Tween-20 in PBS for 10 min and then blocked in 5% bovine serum albumin in PBS at room temperature for 30 min. Routine immunocytochemical characterization was carried out with a panel of antibodies, including anti-fibroblast surface protein (FSP), anti-fibroblast activation protein (FAP), anti-α-smooth muscle actin (SMA), anti-vimentin, anti-fibronectin, and anti-pan-cytokeratin. The primary antibody incubation was performed overnight on a shaker at 4 °C, followed by three washes with PBS. Incubation of fluorescence dye-conjugated secondary antibodies of appropriate species was conducted at room temperature for 2 h, followed by washes and nuclear counter-staining with 0.1 µg/mL 4′-6-diamidino-2-phenylindole (DAPI) (Biotium, Fremont, CA, USA). All fluorescence images were obtained with the EVOS fluorescence microscope (Thermo Fisher Scientific) using 10× objectives. Antibodies used for characterization are listed in Supplemental Table S2.

### 2.8. RNA Isolation and cDNA Synthesis

RNA was extracted from 0.3 x 10^6^ cells using the RNeasy Plus Mini Kit (Qiagen, Toronto, ON) and the quality of the total RNA was assessed on an Agilent 4200 TapeStation System using RNA ScreenTape System (Agilent Technologies, Waldron, Germany) as directed by the manufacturers. Samples utilized for reverse transcription had values of RNA integrity number above 8. RNA purity and concentrations were measured with the ND-1000 Nanodrop Spectrophotometer (Thermo Fisher Scientific).

cDNA was synthesized from 100 ng of total RNA using iScript Reverse Transcription Supermix (Bio-Rad, Hercules, CA) on a BioRad T100 Thermal cycle instrument. The following temperature profile was applied: 25 °C for 5 min, 46 °C for 20 min, and 95 °C for 1 min. The cDNA samples were diluted to 100  ng/μL in nuclease-free water and stored at −20 °C until the quantitative real-time Polymerase Chain Reaction (qPCR).

### 2.9. Quantitative Real-Time Polymerase Chain Reaction

qPCR experiments were conducted in accordance with the Minimum Information for Publication of Quantitative Real-Time PCR Experiments (MIQE) guidelines [25]. BioRad PrimePCR primers (Bio-Rad, Hercules, CA) were used to detect targeted mRNA expressions of the following genes: *FAP*, fibronectin (*FN1*), glyceraldehyde 3-phosphate dehydrogenase (*GAPDH*), Thy-1 cell surface antigen (*THY1*), and vimentin (*VIM*). The primers used for each gene are listed in Supplemental Table S3.

Briefly, the qPCR procedure was carried out on a BioRad CFX Connect Real-Time PCR Detection System. Samples were prepared in a 20-μL reaction volume containing 2 μL of total cDNA, 1X primers, and 1X SsoAdvanced Universal SYBR Green Supermix (Bio-Rad). No-template control and reverse transcription control were included in each assay. All samples were analyzed in duplicate on the same 96-well PCR plate. The thermal cycling was initiated at 95  °C for 2 min, followed by 40 cycles of amplification (95 °C for 5 s and 60 °C for 30 s). Melting curve analysis was performed immediately at 65 °C to 95 °C, in increments of 0.5 °C per 5 s. The threshold cycle (Ct) values <30 were considered as positive expression and Ct values > 30 were considered as negative expression.

The GAPDH mRNA expression from individual samples was used for normalization to obtain delta threshold cycle (ΔCt) values, as this gene expression is stable in fibroblasts [26]. The ΔCt was calculated by subtracting Ct of target gene with Ct of housekeeping gene (*GAPDH*) in an individual sample.

### 2.10. DNA Extraction and Apolipoprotein E (APOE) Genotyping

APOE genotype was determined from the DNA extracted from 0.3 × 10^6^ of P3 fibroblasts using the DNeasy Blood & Tissue Kits (Qiagen) according to the manufacturer’s instructions. The extracted genomic DNA concentration and quality were assessed by the ND-1000 Nanodrop Spectrophotometer (Thermo Fisher Scientific). Samples were diluted to 10  ng/μL in nuclease-free water before storing at −20 °C until genotype analysis.

APOE genotyping was carried out by allele-specific qPCR methodology based on a published protocol with modification [27]. Briefly, 15 μL of reaction volumes were prepared to contain 1X TaqMan Genotyping Master Mix (Thermo Fisher Scientific), 0.5 μM of each allele-specific APOE primers and APOE probes, 0.1 μM of each *ACTB* (actin, beta) primer and probe, and 50 ng of genomic DNA. A no-template control was included in each run. All samples were run in duplicates on the same 96-well PCR plate to reduce the possible inter-run variations. The thermal cycling was initiated enzyme activation at 95 °C for 10 min, followed by 40 cycles with denaturation at 95 °C for 15 s, and anneal/extension at 64 °C for 1 min. APOE genotype of the samples was determined by the ΔCt values [calculated by subtracting the Ct value of beta-actin from the Ct value of three different alleles of APOE (Ɛ2, Ɛ3, and Ɛ4)]. The ΔCt values of the Ɛ2/Ɛ3/Ɛ4 reaction <5 were considered as positive and ΔCt values ≥ 10 were considered as negative.

### 2.11. Generation and Characterization of Human Pluripotent Stem Cells

Expanded fibroblast cultures were passaged to 12-well plates and incubated with Sendai virus (SeV; CytoTune^®^-iPS 2.0 Reprogramming Kit (Thermo Fisher Scientific) at a multiplicity of infection (MOI) of 5. Cells were then passaged onto Matrigel (BD Biosciences, Bedford, MA) in Essential 8TM (E8) medium (Thermo Fisher Scientific). Three-weeks post-transduction single hiPSC colonies were manually picked with a P200 pipette in a manner similar to previously described [28] and passaged onto Matrigel in the presence of E8 medium. HiPSCs were routinely passaged using Accutase (Thermo Fisher Scientific) in mTeSR1 (Stemcell Technologies) supplemented with 5 μM Y-27632 (Tocris Bioscience, Ellisville, MO, USA).

For immunofluorescence characterization, cells were washed twice with PBS and then fixed for 20 min at 4 °C with Cytofix Fixation Buffer (BD Biosciences). Subsequently, cells were washed twice with PBS and permeabilized with Phosflow Perm Buffer III (BD Biosciences) for 20 min at room temperature. After washing twice with PBS, cultures were incubated with primary antibodies overnight and washed twice with PBS. Cultures were then incubated with secondary antibodies at room temperature for 1 h prior to staining for DNA with Hoechst 33342 (2 µg/mL; Thermo Fisher Scientific) for 10 min at room temperature. Imaging was performed on a Nikon Ti-Eclipse inverted microscope with an LED-based Lumencor SOLA SE Light Engine using a Semrock bandpass filter.

For flow cytometry-based characterization, cells were dissociated with Accutase and resuspended at a maximum concentration of 2 × 10^6^ cells in PBS. Cells were stained for 1 h in one test volume of antibody on ice, washed twice, and resuspended in PBS. Cells were passed through a 40-µm cell strainer on an ACCURI C6 (BD Biosciences).

For genotyping, genomic DNA was prepared from expanded clones using the DNeasy kit (Qiagen) and PCR products were generated with Phusion High-Fidelity Polymerase (New England Biolabs, Beverly, MA, USA) with cycling conditions: 98 °C for 30 s, followed by 25 cycles at 98 °C for 10 s, 69 °C for 30 s, and 72 °C for 30 s, followed by a final 10 min 72 °C extension using the following primers: Forward: 5′-GGACGAGACCATGAAGGAGTTGAAGGC -3′, Reverse: 5′- CCACCTGCTCCTTCACCTCGTCCAG -3′. After PCR, amplicons were purified using the QIAquick PCR purification kit (Qiagen) according to the manufacturer’s instructions prior to Sanger sequencing (Genewiz).

To determine if cells were free from SeV reprogramming factors, RNA was isolated from the cells passaged 5 times (NucleoSpin RNA Isolation Kit, Macherey-Nagel) and reverse transcribed using iScript Reverse Transcription Supermix (Bio-Rad). RT-PCR was performed using the following cycling conditions: a 2 min gradient to 95 °C followed by 20 cycles at 95 °C for 5 s and 60 °C for 30 s using the following primers—Forward: 5′ GGATCACTAGGTGATATCGAGC-3′, Reverse: 5′- ACCAGACAAGAGTTTAAGAGATATGTATC-3′. The resultant products were run on a 1% (*w*/*v*) agarose gel.

For tri-lineage differentiation of hiPSCs, cells were dissociated using Accutase and plated as embryoid bodies (EBs) on ultra-low attachment plates in mTeSR1 supplemented with 5 µM Y-27632. After 48 h, the media was changed to differentiation medium (DM) (DMEM/F12, 20% FBS, 1% Penicillin/Streptomycin). After 5 days, EBs were plated on Matrigel-coated plates and cultured with DM. After a minimum of 14 days of differentiation, cells were fixed, permeabilized, and stained for germ layer markers.

For karyotypic analysis, 20 metaphase cells were analyzed using standard protocols for G-banding (Cell Line Genetics, Madison, WI, USA).

### 2.12. Statistical Analysis

This study collected demographic, APOE genotype, clinical, and pathological data as well as PMI, TPI, duration of explant to cell outgrowth (S1), and duration of cell outgrowth to cryoprotection (S2) from autopsy cases. The means and standard deviations (mean ± S.D.) were calculated for each of the quantitative parameters. The GAPDH gene normalized ΔCt values for each gene were used for assessing the abundance of mRNA levels. Statistical differences between two groups were analyzed by unpaired, 2-tailed *t*-tests. The assumption of equal variance was determined prior to testing for the significance of the group differences. If equal variances were rejected, the Welch-test was used for determining the significance of the difference. If more than two groups were evaluated, the groups were analyzed by one-way ANOVA with Dunnett or Tukey test. Pearson’s or Spearman’s correlation analyses were performed to determine the relationship between demographic, clinical, and pathological features, PMI, TPI, and S1 and S2. A *p*-value ≤ 0.05 was considered as statistically significant. All analyses were performed using MedCalc statistical software v. 19.1 (MedCalc Software Ltd., Ostend, Belgium).

## 3. Results

### 3.1. Neuropathological Features of the Donors

**Figure 2:**
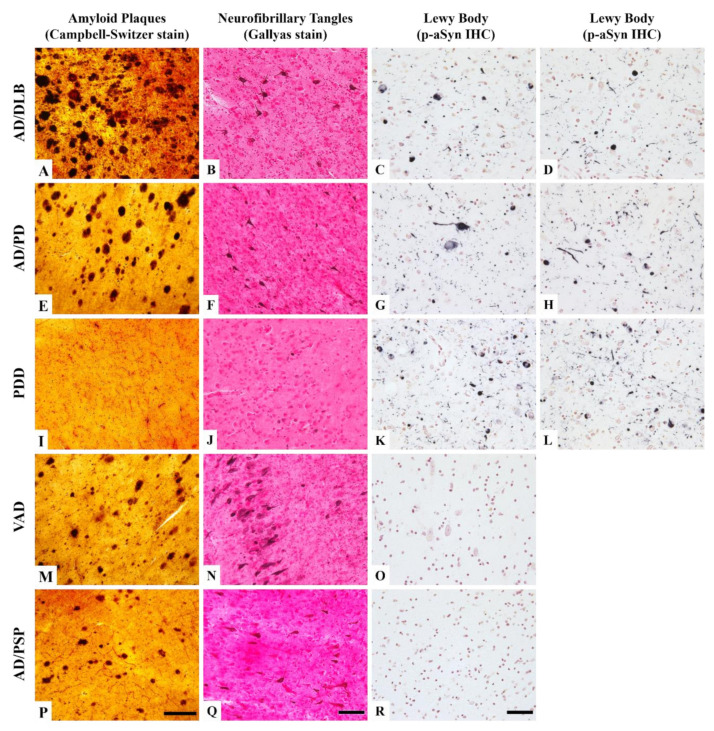
Representative staining profiles of various neuropathological diagnoses in Campbell-Switzer and Gallyas staining and immunohistochemical staining of p-αSyn. The photo images were taken from a case with AD/DLB diagnosis (**A**–**D**), a case with AD/PD diagnosis (**E**–**H**), a case with PDD diagnosis (**I**–**L**), a case with VAD (**M**–**O**), and a case with AD/PSP (**P**–**R**). (**A**,**E**,**I**,**M**,**P**): Amyloid plaques in dark brown color in various abundance are shown in the temporal cortical area. (**B**,**F**,**J**,**N**,**Q**): Neurofibrillary tangles in medium brown are shown at various densities in the entorhinal cortex. (**C**,**D**,**G**,**H**,**K**,**L**,**O**,**R**): Lewy body and Lewy neurites in black are shown in neutral red counter stained amygdala. Scale bars: Campbell-Switzer stain, 250 μm; Gallyas stain, 150 μm; p-αSyn immunohistochemical stain, 50 μm. Abbreviation: AD, Alzheimer’s disease; DLB, dementia of Lewy body; PD, Parkinson’s disease; PDD, Parkinson’s disease with dementia; VAD, vascular Alzheimer’s dementia; p-αSyn, phosphorylated α-Synuclein; PSP, progressive supranuclear palsy.

Twenty of the 29 autopsy cases in this study had neuropathological diagnoses completed after cells were banked. The pathological characterization was based on a semi-quantitative assessment of 5 brain regions for AD pathology and 10 brain regions for LB pathology. The results showed that most of the donors had more than one neurodegenerative disease. Examples of neuropathological features from parahippocampal, entorhinal, and amygdala areas in some of the cases are shown in Figure 2. In an AD and dementia with Lewy Body Disease (AD/DLB) brain and AD with PD brain, frequent amyloid plaque density in the temporal cortex, intermediate tangle density in the para-hippocampal area, and abundant LB pathology in the amygdala were observed (Figure 2A–H). A PD with dementia (PDD) donor has no amyloid plaque and low tangle density but abundant LB pathology (the sum of LB density was 36 out of a total score of 40) (Figure 2I–L). Figure 2M–O show a vascular dementia with AD (VAD) case exhibiting frequent amyloid and tangle density but had no LB pathology. Lack of LB pathology was also found in an AD with progressive supranuclear palsy (AD/PSP) case, but the densities of amyloid plaques and tangles were lower than the VAD case (Figure 2P–R).

There were 6 cases whose pathology in the brains did not meet the diagnosis criteria of AD or α-synucleinopathy. However, three of them were clinically diagnosed with mild cognitive impairment (MCI). As final diagnosis was based on both clinical-neuropathological criteria, these three donors were not classified as NC. We had one amyotrophic lateral sclerosis (ALS) case with a C9orf72 gene mutation and one multiple system atrophy (MSA). Both had no LB pathology. The ALS donor had little aging-associated AD pathology in the brain. The rest of the cases which await pathological diagnosis to be completed were clinically diagnosed as PD (N = 3), AD (N = 2), and NC (N = 4).

### 3.2. The Explant and Cell Cultures

**Figure 3:**
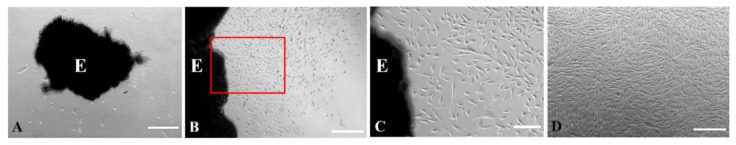
Phase-contrast images of cell migration and expansion from explants. (**A**). The dark area is a dermal explant (E) in 6-well plate at 6-day old culture. 4× objective. This figure shows the initial cell migration out of explants. (**B**). From the same case, at 11th day more cells were present outside the explant at the same magnification. The red inset area is shown at higher magnification in C. (**C**). Cells exhibited elongated spindle shape cell bodies. 10× objective. (**D**). A representative image of fibroblasts reaching confluence in P1 subculture. Scale bars in A, B, and D represent 500 µm and in C 200 µm.

The progress of the culture could be observed from two stages: stage 1 (S1), from explant plating to the time that cell outgrowth was first observed, and stage 2 (S2), from first cell outgrowth to first cryogenic storage of the P3 cells. The length of time for the explants to attach varied from case to case; ranging from less than 24 h to two weeks. Once attached, the shape of explant tissues was gradually flattened, and the color lightened. Figure 3 illustrates the progression from explant culture to cell culture from a representative case. Cells migrated out of explants into the empty space in the culture wells within 5–38 days (median: 11.5 days). Patches of epidermal keratinocytes could appear first in some of the explant cultures. In the following two weeks, the cell population of mixed morphology was gradually taken over by the cells with a spindle shape (Figure 3C) and reached confluency (Figure 3D).

For subculture, cells at 80–85% confluence were detached from original wells to expand to P1 at 1:2 growth area ratio; the expansion continued until P3, a stage at which more than 5–30 million cells could be produced. At P3, cells usually formed a monolayer, exhibiting the characteristic morphology of dermal fibroblasts. Cell bodies of the fibroblasts were in elongated spindle shape while the cytoplasm appeared dense and opaque. Cells aligned in parallel with each other without piling up.

### 3.3. Immunofluorescence Characterization of P3 Fibroblasts

**Figure 4:**
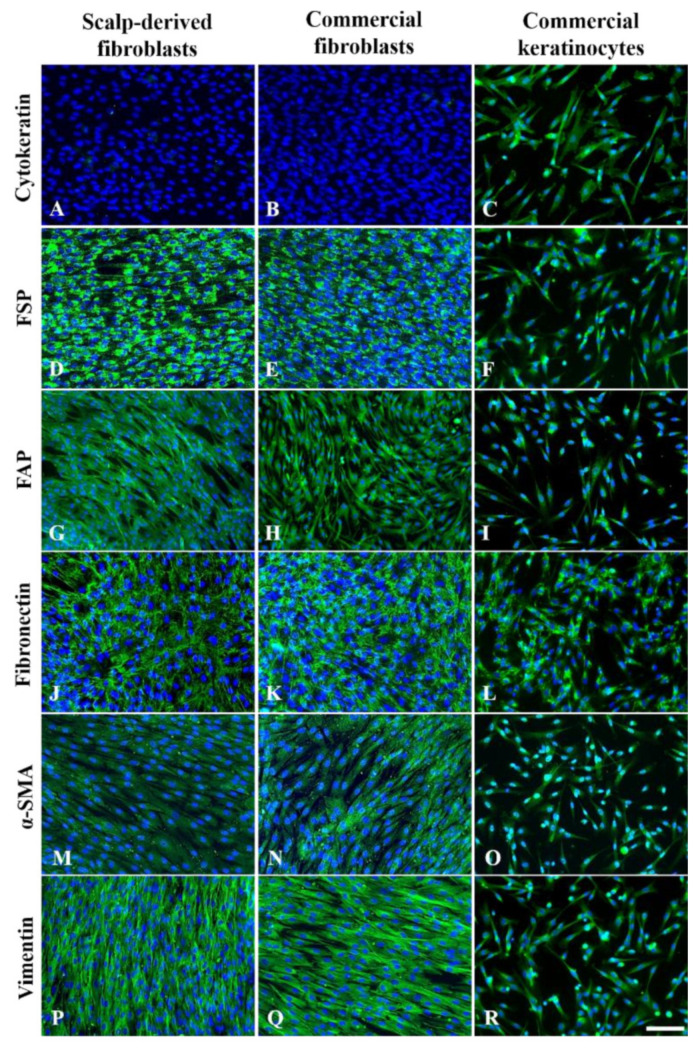
Representative expression profiles of various markers in postmortem scalp-derived fibroblasts, commercial dermal fibroblasts, and commercial keratinocytes. The presence of green immunofluorescence shows the positive staining of the markers: (**A**–**C**), cytokeratin; (**D**–**F**), FSP (fibroblast surface protein); (**G**–**I**), FAP (fibroblast activation protein); (**J**–**L**), fibronectin; (**M**–**O**), α-SMA (α-smooth muscle actin); (**P**–**R**), vimentin. The nuclei of cells are shown in blue fluorescence by DAPI dye. The top row shows the presence of cytokeratin immunoreactivity in keratinocytes but not in fibroblasts. All other markers are all positively detected in the postmortem scalp-derived and commercial fibroblasts. Scale bars represent 100 μm.

We used a panel of antibodies to detect the proteins frequently used for characterizing fibroblasts, including fibroblast markers FSP and FAP; extracellular protein fibronectin, and cytoskeletal proteins α-SMA and vimentin. As keratinocytes could be co-isolated in the explant scalp culture, an antibody for pan-cytokeratin was used to detect the presence of keratinocytes in P3 cell culture. The results are shown in Figure 4.

### 3.4. Characterization of Fibroblast Cultures by qPCR Analysis

**Figure 5:**
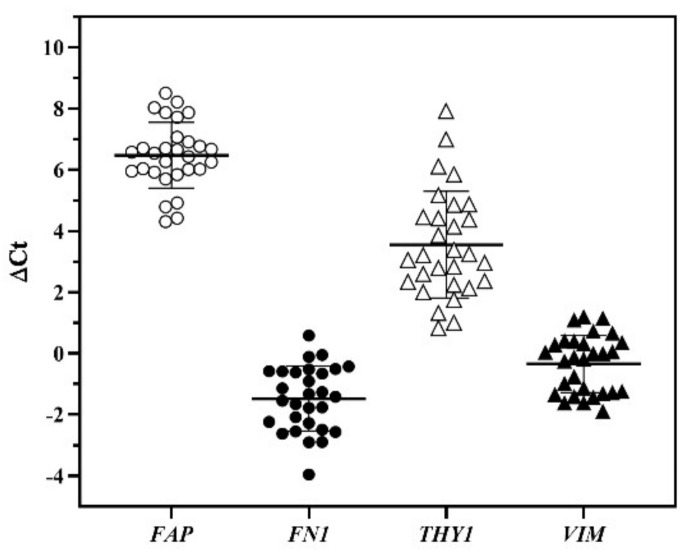
The expression of mRNA levels indicated by ΔCt values. The horizontal bars represent mean ± standard deviation from 29 cases. The symbols denote different genes. *FAP*: fibroblast activation protein; *FN1*: fibronectin 1; *THY1*: Thy-1 cell surface antigen; *VIM*: vimentin.

As qPCR is a sensitive technique for gene expression analysis, we explored the utility of a 6-gene panel for the purpose of routine characterization of the banked cells. These genes were selected for the significance in cellular property and functions, including FAP, FN1, THY1, and VIM, along with the housekeeping gene GAPDH and keratinocyte gene KRT14. KRT14 gene was used to detect the presence of the keratinocytes in the banked cells. In general, a Ct value ≥ 30 was considered below the sensitivity of detection. The Ct values of keratinocyte marker KRT14 in majority of our banked cases were at an undetectable level. There were only two cases that had Ct < 30, indicating the co-presence of keratinocytes in these cases. The ΔCt value from each gene was obtained by subtracting the GAPDH Ct value from the Ct value of the gene of interest. The distribution of the ΔCt values for the four genes are shown in Figure 5. The banked cells from 26 cases had FN1 ΔCt values below zero, meaning that their FN1 mRNA was more abundant than the levels of GAPDH. There were 18 cases whose VIM levels were expressed more abundantly than GAPDH, based on negative values of ΔCt. The ΔCt values of THY1 and FAP genes from all cases were all higher than GAPDH Ct values, indicating lower expression than GAPDH gene.

### 3.5. Fibroblast Gene Expression and Culture Time Course

We explored whether durations of the S1 and S2 stages in culture were affected by the delay of autopsy (PMI) or delay of tissue processing for culture (TPI). We found that neither PMI nor TPI had any effect on S1 or S2. The results indicated that these postmortem factors did not affect the culture duration. We also did not detect any association between S1 and S2.

We then explored whether there was any association of the duration of S1 or S2 with the gene expression of P3 cells using ΔCt values by Spearman’s analysis. Interestingly, we detected a positive correlation between FN1 ΔCt and S1 (ρ = 0.444, *p* = 0.0180), indicating that the longer the cells took to grow out of explants, the larger the FN1 ΔCt values, indicating lower FN1 expression) in the P3 cells. Similar results were found in VIM ΔCt with S1 (ρ = 0.430, *p* =0.0222). None of these genes correlated with S2. Nevertheless, THY1 ΔCt had a negative correlation with S2 (ρ = −0.463, *p* = 0.0132, N = 29), indicating the association of longer S2 with higher THY1 mRNA level in P3 cells. 

### 3.6. Quality Control for Cell Banking

As part of quality controls, we verified the positive staining and mRNA expressions with a commercial source of human dermal fibroblasts. When the ΔCt values of each gene were computed, we found that the values from the commercial fibroblast line fell within the range of the fibroblasts prepared from the donors in this study.

Also, we assessed the frequency of keratinocytes present in our P3 fibroblast cultures. KRT14, an intermediate filament protein, is one of the keratins expressed by keratinocytes present in the proliferative basal epidermal layer. In 29 cases, there were only two cases that contained low levels of KRT14 mRNA, indicated by the Ct values of 26 and 29 in these two cases**.** The mRNA levels of FAP, FN1, THY1, and VIM in these two cases were comparable to the fibroblasts that were negative for KRT14 mRNA. Moreover, the commercial keratinocyte expressed lower levels of all 4 genes than these two mixed cell-type cases. Taken together the evidence, fibroblasts were the predominant cell type even when the P3 culture contained keratinocytes.

The aliquots of cryoprotected P3 fibroblasts stored in liquid nitrogen were tested for the yield of viable cells after defrosting. The revival rates defined by the percentage of live cell counts in total cell numbers were >95%, and cells attached and proliferated within 24 h. The presence of the fibroblast markers was consistent with the dermal fibroblasts obtained from two commercial sources.

### 3.7. Generation of hiPSCs from Patient Fibroblasts

**Figure 6:**
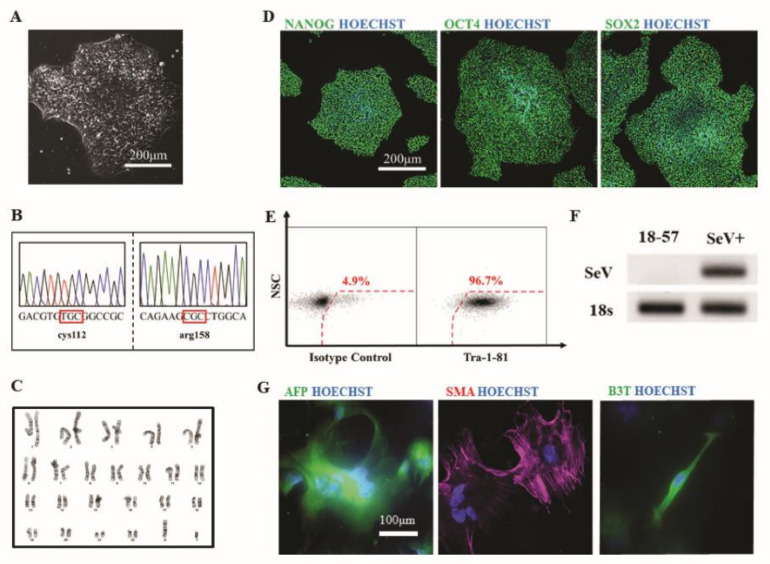
Characterization of human induced pluripotent stem cells (hiPSCs) derived from scalp fibroblast cultures. (**A**). Phase contrast image of a representative clone. (**B**). Sanger sequencing analysis of exon 4 of the APOE gene confirms homozygosity for the e3 allele. (**C**). Karyotype analysis of a representative clone reveals a normal male euploid karyotype. (**D**). Immunofluorescence staining for pluripotency markers NANOG, OCT4, and SOX2. (**E**). Flow cytometry analysis of pluripotency-related marker TRA-1-81. (**F**). RT-PCR analysis demonstrates absence of SeV transgenes in expanded hiPSCs. Sendai virus infected fibroblasts were used as a positive control. (**G**). Immunofluorescence staining of embryoid bodies for markers associated with endoderm (AFP), mesoderm (SMA), and ectoderm (B3T). Nuclei were counterstained in blue color.

To provide proof-of-principle that the human autopsy-derived scalp fibroblasts could be used to generate hiPSCs, we used a non-integrating Sendai viral approach to reprogram one of the established patient fibroblast lines (line #2). Several clones were isolated and subjected to detailed phenotypic characterization. All clones examined had characteristic pluripotent stem cell morphology (Figure 6A). Sanger sequencing analysis of hiPSCs at the APOE locus confirmed homozygosity for the Ɛ3 genotype, consistent with the parental fibroblasts (Figure 6B). Expanded hiPSCs had a normal euploid karyotype (Figure 6C). In addition, immunofluorescent (Figure 6D) and flow cytometry (Figure 6E) analysis revealed all undifferentiated clones expressed high levels of markers associated with the undifferentiated, pluripotent state, such as NANOG, OCT4, SOX2, and TRA-1-81. The absence of reprogramming viral transgenes in expanded hiPSC was confirmed by RT-PCR (Figure 6F). Finally, to verify tri-lineage differentiation potential, hiPSCs were differentiated in vitro using an embryoid body (EB)-based protocol. Immunofluorescent analysis of plated EBs demonstrated the presence of cells representative of endoderm (measured by expression of alpha-fetoprotein, AFP), mesoderm (measured by expression of smooth muscle actin, SMA), and ectoderm (measured by expression of beta-3 tubulin, B3T) (Figure 6G). Collectively, this analysis reveals that patient fibroblasts can be fully reprogrammed to hiPSCs.

## 4. Discussion

### 4.1. Diverse Patient Phenotypes

In this report, we described the development of an autopsy-derived human scalp fibroblast banking program at BSHRI. During 19 months of time, we successfully cultured scalp fibroblasts from 29 autopsy cases of different ages, APOE genotypes, and disease diagnoses. The neuropathological examination from 20 cases revealed diversity within each major classification of sporadic AD or PD, of which clinical assessment alone could not achieve. Among these neuropathologically diagnosed cases, except for two AD and one PD case that had no co-presence of other major neurodegenerative diseases, 6 patients met diagnostic criteria for both AD and diseases like DLB, PSP, and PD. Additionally, we have also banked cells from patients with MSA, ALS, VAD, and PD with dementia and NC, the subjects without neurological diseases. Approximate 33% of the BBDP participants had no clinically apparent neurological diseases before they died. After confirmation from neuropathological examination for not meeting any criteria for disease diagnosis, they are classified as NC. In the current cohort, three of such cases had clinical diagnosis of MCI and the other three were cognitively normal. Cells derived from the cognitive and neuropathologically normal cases could be used as controls for cells derived from diseased cases. Our cell banking program has long-term potential, as over time, there will be an accumulation of the number in each disease phenotype as well as in the number and types of specific genotypes for research. Ultimately we plan to have next-generation sequencing genomic results available for all subjects.

### 4.2. The Features of the Culturing Procedure

Fibroblasts can be obtained from different parts of the body. Scalp tissues were chosen for this program because of its accessibility at autopsy procedure. Scalp samples are easily obtained 10 min into the autopsy procedure. The scalp also contains less adipose tissue, which could reduce the time for trimming, when compared to abdominal skin. We chose the approach of explant culture over dissociated cell culture, avoiding the breakup of extracellular matrix and alteration of cell-surface properties caused by enzymatic treatment. Although skin tissue is wounded by dissection and mincing, the injury may release molecular cues for skin wound response [29,30]. Cell migration towards injured sites is a characteristic of wound response. The response would likely depend on cellular and tissue vitality during postmortem conditions which are affected by antemortem and perimortem factors. The attachment of explants to plastic culture plates was an essential step towards successful cell outgrowth. Unattached explants were discarded after 6 weeks. Occasionally, attached explants could fail to have cells migrating out. We estimated that 30% of autopsy cases were unsuccessful due to a combination of factors that we were not able to identify. Age alone was not a factor affecting the success in our procedure, as the age range of successful cases was 43–101 years old. None of the processing delays (PMI or TPI) or APOE genotypes were determinants of successful culture.

Our explant tissues contained mainly papillary (adjacent to the epidermis) and reticular dermal layers and hypodermis. Anatomically, it is inevitable for the explants to also contain variable amounts of hair follicle unit, microvessels, and follicular epidermis. Having these companion structures in explant could be more beneficial than not, as an environment sustaining cell viability could be maintained. The growth factors and cytokines could be available in the extracellular matrix and/or secreted by stem cells and progenitor cells residing in the explant [31,32,33].

By plating 50–60 pieces of dermal fragments in 5 or 6 of 6-well plates from one autopsy case, the current workflow could generate cryogenic fibroblasts in 8 weeks. On average, the S1 stage takes 13 days (range: 5–38 days). The entire culture process takes about two months. Interestingly, the duration coincided with a previous report of scalp-derived fibroblasts from 146 subjects who had a mean age of 45.3 ± 15.7 years [34]. Their starting materials were 8-inch^2^ scalp which generated 5 to 10 million subcultured cells. The sample size in our procedure was smaller (approximately, 3.1 inch^2^), and 5 to 30 million P3 cells were generated.

The use of postmortem human tissues to culture adult fibroblasts is not new. Previously, skin samples of various anatomical regions had been used to culture fibroblasts: abdomen [35]; forearm, leg, and torso [36], leptomeninges [37], scalp and dura mater [34], archived non-cryoprotected dura mater [38], abdomen, scalp, lung, vocal fold, soft palate, trachea, and upper Gingiva [39]. Notably, the epigenetic profiles that could potentially affect the efficiency of iPSCs reprogramming and subsequently differentiation could vary by anatomical regions. Scalp fibroblasts displayed more inter-individual differences in epigenetic profiles than fibroblasts from the dura mater, possibly due to cumulative exposure and response to the environment [40]. Although fibroblast culture has reportedly been successful from between 1 to 11 years after archiving human frozen tissues, as expected the success rate with longer storage was reduced (to 44%) and longer-preserved cultures had a higher frequency of contamination issues [38]. Our workflow was developed to complete processing and banking within two to three months after a patient died, reducing postmortem factors that might alter the ability of cell expansion and downstream application, such as reprogramming, differentiation, or conversion.

Bliss et al. [34] assessed the production of fibroblasts from dura mater and scalp of a large series of postmortem cases and found that scalp tissue achieved higher cell yield than dura mater due to a higher proliferating rate. The mean age of their scalp donors was 45.3 ± 15.7 years at a mean PMI of 31.7 ± 14.1 h. They processed tissues immediately after they received them. The BBDP participants are at an older age; in our study, the mean age was 82.5 ± 12.4 years, and PMI was 4.0 ± 1.4 h though the TPI was 14.6 ± 9.8 h. The longer TPI came from autopsy cases that died during the weekend. Nevertheless, the tissue samples were kept in the special media. While our procedure removed the epidermis layer by dissection, this group removed epidermis by 24-h Dispase II digestion. Without enzymatic digestion, we had shortened the entire procedure to less than two hours. It is possible that a shorter procedure could maintain cell viability, thereby success rate. Bliss et al., [34] reported 55% success, whereas our success rate was 70%.

A recent study focused on obtaining fibroblasts from PD patients [37]. The group established a procedure from postmortem meninges. Using 6 × 6 cm^2^ (5.6 inch^2^) meninges, 20–30 million fibroblasts were produced in 6–8 weeks. These were from 70–88 years old PD patients with 8–31 years of PD history and the PMI was between 10–24 h. Cells were positive for the fibroblast marker SERPINH (CD47). Although cells exhibited characteristic morphological features of fibroblasts, it was unclear whether contamination of endothelial and smooth muscle cells could be problematic, because meninges is a blood vessel bed.

### 4.3. The Features of Postmortem Scalp Fibroblasts

Fibroblasts are mesenchymal cells that bear the characteristic of mesenchymal stem cells [41,42]. Up to this date, fibroblasts cannot be defined by any single marker. Thus, we explored a set of genes (FAP, FN1, THY1, and VIM) that have cellular property and functional significance to the fibroblasts for routine characterization of the banked cells.

It is well known that the secretion of extracellular matrix (ECM) proteins is an important function of fibroblasts. Among the ECM proteins, fibronectin plays an important role in extracellular matrix assembling, adhesion, and migration of the cells, and the generation of iPSC [43,44,45]. Cytoskeletal protein VIM modulates motility and morphological structure of fibroblasts [46,47,48]. Fibroblasts share markers of mesenchymal stem cells (MSC), THY1 [39,49]. THY1 is involved in regulating the differentiation of mesenchymal stem cells [38] and has been used to select fibroblasts [50,51]. In pairing with FAP, Korosec A et al. [52], identified reticular fibroblasts to be FAP^−^CD90^+^ and papillary fibroblasts to be FAP^+^CD90^−^. FAP was also included in our gene panel. FAP protein is a unique post-proline protease, upregulated in activated fibroblasts and functioned in tissue remodeling, tumorigenesis, fibrosis, energy metabolism, and blood coagulation [53,54,55].

We found that these four genes were positively expressed by all our P3 cells and commercial dermal fibroblasts cultured in the same media. Importantly, we also analyzed the expression of a keratinocyte gene, KRT14, in our P3 cells to assess the extent of co-presence of keratinocytes in our culture. Although low levels of KRT14 gene were detected in two banked cases, the four genes were still expressed at the levels comparable to the fibroblasts negative for KRT14 gene. Taken together, we were able to use the positively and negatively expressed gene panel along with morphological features to routinely characterize the banked cells.

The ΔCt values revealed that these genes were variably expressed: relatively speaking, the levels were highest for FN1 and VIM, moderate for THY1, and low for FAP. Interestingly, when correlation analysis was performed, we found the link of mRNA expression levels of three genes with the stages of culture course; namely, longer S1 duration (time for cell outgrowth from explant) was associated with lower levels of FN1 and VIM mRNA, while shorter S2 duration associated with higher THY1 expression. THY1 levels have been shown to affect pluripotency and differentiation of MSC, depending on the origin of fibroblasts and the context of activities [56,57]. Currently, we do not know whether these findings reflect intrinsic property of the tissues and/or the effects of premortem factors.

### 4.4. Future of the Human-Derived Fibroblast Banking

Research using differentiated cells from fibroblasts reprogrammed to hiPSCs lines has played an important role in unraveling mechanisms for neurodegenerative disease onset and progression [58,59,60]. Here, we provide proof-of-principle that human postmortem scalp fibroblasts can be reprogrammed to hiPSCs. These hiPSCs display characteristic hiPSC morphology, a normal euploid karyotype, high expression of key pluripotency markers, and tri-lineage differentiation potential. Success from using postmortem abdominal fibroblasts to obtain hiPSC has been reported previously [36]. More recently, direct reprogramming technology without reprogramming fully to the pluripotent state allows better preservation of age-, disease-, and environment-induced epigenetic signatures [61]. As such, there have been successful procedures using human-derived fibroblasts to generate astrocytes and neurons [62,63,64,65]. In the future, the fibroblast lines developed as part of this study could be employed in concert with direct reprogramming strategies to generate human neural cell models that could better recapitulate the genomic and epigenomic features specific for the diseases.

Uses of primary fibroblasts and fibroblast lines have also detected phenotypic features and functions differentiating between subjects with or without gene mutation, and non-diseased controls [12,66,67]. Fibroblasts derived from AD and PD patients could also replicate the mitochondrial defective mechanisms [17]. As for ALS, the comparison between biopsied skin fibroblasts derived from sporadic ALS, primary lateral sclerosis, C9 mutated ALS, and controls were shown to exhibit differential biogenic functions [68]. The gene expression profiles involved in RNA processing, hypoxia response, and metabolism were also different in ALS patients [69]. DNA defects in repair exhibited in motor neurons could also be recapitulated from the fibroblasts derived from sporadic ALS cases [70]. In the near future, we will be able to conduct such comparative studies when cells from more cases are banked.

## 5. Conclusions

We have reported successful banking of fibroblasts cultured from well-characterized human research subjects with sporadic or familial neurodegenerative diseases. The initial gene expression survey has shown a positive expression of the genes that are related to the functions of the fibroblasts. Continuing efforts in this human fibroblast repository program will lead to the accumulation of a large number of diverse fibroblast lines in the next few years. The goal of this cell repository program is to facilitate the widespread use of these human-derived cells to develop models that recapitulate genomic and epigenomic features of the sporadic and familial neurodegenerative diseases.

## Figures and Tables

**Table 1:** Demographics and clinical history of the autopsy cases.

Variables, N	Mean ± S.D. and Ratios	Minimum–Maximum
Age, N = 29	83.6 ± 12.2 years	43–101 years
Gender, N = 29	14 males:15 females	
APOE genotypes, N = 29	2/3;3/3;3/4;4/4 = 3:19:6:1	
Years since diagnosis, N = 21	9.0 ± 6.6 years	1–24 years
Age at diagnosis, N = 21	71.4 ± 15.1 years	37–91 years
Years of dementia, N = 13	4.5 ± 4.0 years	1–14 years
Age of dementia onset, N = 12	77.4 ± 10.6 years	58–92 years

Abbreviations: N: number of patients; S.D.: standard deviation; APOE: Apolipoprotein E.

**Table 2:** Measures of last memory and movement functions in relation to completed neuropathology for 20 donors.

Final Diagnosis	MMSE (N)	UPDRS (N)	Sum * of Plaque Density Scores (N)	Sum * of Tangle Density Scores (N)	Sum ^#^ of LB Density Scores (N)
NC	26.8 ± 3.5	12.2 ± 13.4	7.8 ± 5.3	6.8 ± 2.9	2.5 ± 6.1
	(6)	(6)	(6)	(6)	(6)
AD	5.5 ± 0.7	30.5 ± 31.8	14.3 ± 1.1	12.0 ± 0.7	14.0 ± 1.4
	(2)	(2)	(2)	(2)	(2)
AD/DLB	5.5 ± 7.8	57.5 ± 31.8	14.5 ± 0.7	10.8 ± 6.0	35.5 ± 0.7
	(2)	(2)	(2)	(2)	(2)
AD/PSP	11.0 ± 8.5	14.0	11.3 ± 1.8	8.3 ± 0.4	0.0
	(2)	(1)	(2)	(2)	(2)
AD/PD	9.0	61.0	14.5 ± 0.7	5.3 ± 0.4	37.5 ± 2.1
	(1)	(1)	(2)	(2)	(2)
VAD	22.0	12.0	12.5	15.0	0.0
	(1)	(1)	(1)	(1)	(1)
PD	29.0	28.0	2.5	5.5	29.0
	(1)	(1)	(1)	(1)	(1)
PDD	NA	NA	0.0 (1)	5.0 (1)	36.0 (1)
ALS	23.0 (1)	NA	0.0 (1)	0.5 (1)	0.0 (1)
MSA	29.0 (1)	NA	11.0 (1)	6.5 (1)	0.0 (1)
DNOS	29.0	34.0	0.0	3.0	0.0
	(1)	(1)	(1)	(1)	(1)

* Sum maximum is 15. ^#^ Sum maximum is 40. Abbreviations: NC: normal controls, AD: Alzheimer’s disease, DLB: dementia of Lewy body, PSP: progressive supranuclear palsy; PD: Parkinson’s disease; VAD: vascular Alzheimer’s dementia; PDD: Parkinson’s disease with dementia; ALS: amyotrophic lateral sclerosis; MSA: multiple system atrophy; DNOS: dementia not otherwise specified; MMSE: Mini-Mental State Examination; UPDRS: Unified Parkinson’s disease rating scale; NA: not available; LB: Lewy body.

## Supplementary Materials

The following are available online at https://www.mdpi.com/2073-4409/9/11/2383/s1, Table S1: sources and concentrations of antibodies used for Immunofluorescence detection in this study, Table S2: the primers used for qPCR in fibroblast characterization, Table S3: characteristics of the patients in this study, Figure S1: representative images of amyloid plaque and neurofibrillary tangle density, Figure S2: representative images of p-αSyn-immunoreactive Lewy body (LB) and Lewy neurites (LN) in amygdala, Figure S3: representative images of p-αSyn-immunoreactive Lewy body (LB) and Lewy neurites (LN) in neutral red counter-stained temporal cortex.
