# Supplementary material for: Human Autopsy-Derived Scalp Fibroblast Biobanking for Age-Related Neurodegenerative Disease Research
Source: Cells. 2020 Oct 30;9(11):2383. doi: 10.3390/cells9112383 (PMC7692621; doi:10.3390/cells9112383)
Supplement: Supplementary file 1 [file cells-09-02383-s001.pdf]

cells

Supplementary Materials

Table S1. *Sources and concentrations of antibodies used for Immunofluorescence detection in this study.*

Antibody	Company	Catalog#	Source	Dilution
Anti-AFP	Santa Cruz	SC-15375	Rabbit IgG Pab	1:50
Anti-B3T	Fitzgerald	10R-T136A	Mouse IgG2a Pab	1:1,000
Anti-pan Cytokeratin	Abcam	AB80826	Mouse IgG Mab	1:2,000
Anti-FAP	OriGene	TA347770	Rabbit IgG Pab	1:1,000
Anti-Fibronectin	Abcam	AB45688	Rabbit IgG Mab	1:2,000
Anti-FSP	Novus Biologicals	NB100-1845	Mouse IgM Mab	1:1,000
Anti-NANOG	Thermo Fisher Scientific	PA1-097	Rabbit IgG Pab	1:200
Anti-SMA	R&D Systems	MAB1420	Mouse IgG2A Mab	1:2,000
Anti-SMA	Santa Cruz	SC-53015	Mouse IgG2B Mab	1:50
Anti-SOX2	Thermo Fisher Scientific	PA1-094	Rabbit IgG Pab	1:200
Anti-TRA-1-81	BioLegend	330706	Mouse IgM Mab	1:30
Anti-OCT4	Thermo Fisher Scientific	PA5-27438	Rabbit IgG Pab	1:200
Anti-Vimentin	Thermo Fisher Scientific	MA-3745	Mouse IgM Mab	1:2,000
Anti-rabbit Alexa 488	Thermo Fisher Scientific	A-11034	Goat IgG Pab	1:2,000
Anti-rabbit Alexa 488	Thermo Fisher Scientific	A-21206	Donkey IgG Pab	1:500
Anti-mouse Alexa 488	Thermo Fisher Scientific	A-11029	Goat IgG Pab	1:2,000
Anti-mouse Alexa 488	Thermo Fisher Scientific	A-21042	Goat IgG Pab	1:2,000
Anti-mouse Alexa 488	Thermo Fisher Scientific	A-21202	Donkey IgG Pab	1:500
Anti-mouse Alexa 647	Thermo Fisher Scientific	A31571	Donkey IgG Pab	1:500
Anti-goat Alexa 488	Thermo Fisher Scientific	A-11078	Rabbit IgG Pab	1:2,000
Alexa 647 mouse IgM Isotype Control	Biolegend	MM-30	Mouse IgM Pab	1:30

Abbreviations: AFP, alpha-fetoprotein; B3T, class III beta-tubulin; FAP, fibroblast activation protein alpha; SMA, smooth muscle actin; FSP, Fibroblast Surface Protein; IgG: immunoglobulin G; IgM: immunoglobulin M; Pab: polyclonal; Mab: monoclonal.

Table S2. *The primers used for qPCR in fibroblast characterization.*

Gene Name	Gene Symbol	Length (bp)	Bio Rad Assay ID
Fibroblast activation protein, α	*FAP*	97	qHsaCID0018575
Fibronectin 1	*FN1*	138	qHsaCID0012349
Glyceraldehyde-3-phosphate dehydrogenase	*GAPDH*	117	qHsaCED0038674
Thy-1 cell surface antigen	*THY1*	120	qHsaCED0036661
Vimentin	*VIM*	82	qHsaCID0012604

Table S3. *Characteristics of the patients in this study.*

No.	Age	Gender	APOE	Years of Dx	Clinic. Dx	Patho. Dx	Sum Plaque	Sum Tangles	Sum LB
1	≥90	F	3/4	11	AD	AD	13.5	11.5	15
2	≥90	M	3/3	3	DNOS	DNOS	0	3	0
3	75	M	3/3	4	PD	PD	2.5	5.5	29
4	76	F	3/4	4	MSA	MSA	11	6.5	0
5	≥90	M	3/3	5	MCI	NC/MCI	9.5	8	0
6	81	F	3/3	2	MCI	NC/MCI	12	6	0
7	84	M	3/3	8	MVD	AD/PSP	12.5	8.5	0
8	80	M	3/4	8	PD	AD/DLB	15	15	36
9	≥90	F	3/3	2	MCI	NC/MCI	14.5	12	0
10	≤60	M	2/3	1	ALS	ALS	0	0.5	0
11	82	M	3/3	7	DLB	AD/DLB	14	6.5	35
12	75	M	3/4	14	PD	PD/AD	14	5.5	36
13	74	M	3/4	24	PD	PD/AD	15	5	39
14	60	M	3/3	23	PDD	PDD	0	5	36
15	≥90	M	3/3	5	VD	VAD	12.5	15	0
16	≥90	F	2/3	10	PD	AD/PSP	10	8	0
17	82	F	3/3	0	NC	NC	1.5	5	0
18	81	F	2/3	8	AD	AD	15	13.5	0
19	76	F	3/3	0	NC	NC	2	5.5	15
20	73	F	3/4	0	NC	NC	7.5	4	0
21	86	F	3/3	20	PD	-	-	-	-
22	≥90	F	3/3	0	NC	-	-	-	-
23	72	M	3/3	12	PD	-	-	-	-
24	88	M	3/3	0	NC	-	-	-	-
25	85	M	3/3	12	PD	-	-	-	-
26	73	F	4/4	10	AD	-	-	-	-
27	≥90	F	3/3	0	NC	-	-	-	-
28	≥90	F	3/3	0	NC	-	-	-	-
29	≥90	F	3/3	8	AD	-	-	-	-

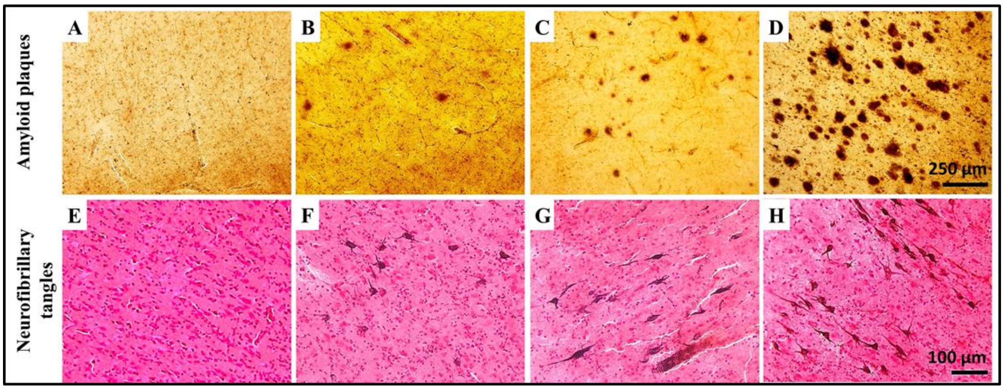

Figure S1. Representative images of amyloid plaque and neurofibrillary tangle density. A–D. Amyloid plaques in brown color are shown from score 0–3 in the inferior temporal gyrus. E–H. Neurofibrillary tangles in dark brown are shown from score 0–3 in the entorhinal cortex. Neural formalin-fixed, 40 µm-thick brain sections were used for our standard procedure for histological processing and staining. Calibration bars are shown in D,H.

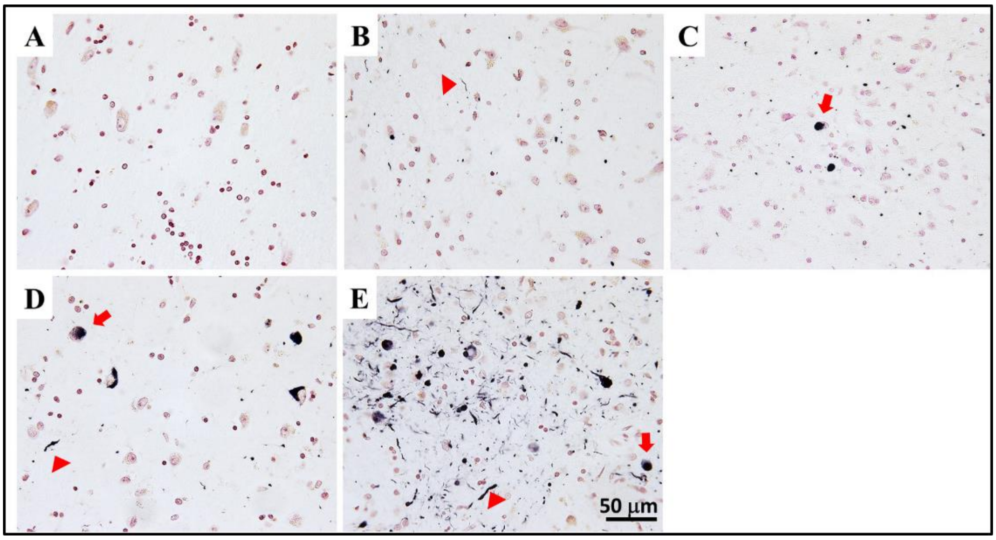

Figure S2. Representative images of p-αSyn-immunoreactive Lewy body (LB) and Lewy neurites (LN) in amygdala. The immunoreactivity was shown in black profiles in the neutral red counter stained tissue sections. From A–E demonstrate LB and LN density from score 0–4. Representative LB profiles are indicated by arrows and LN by arrowhead. Calibration bar is shown in E.

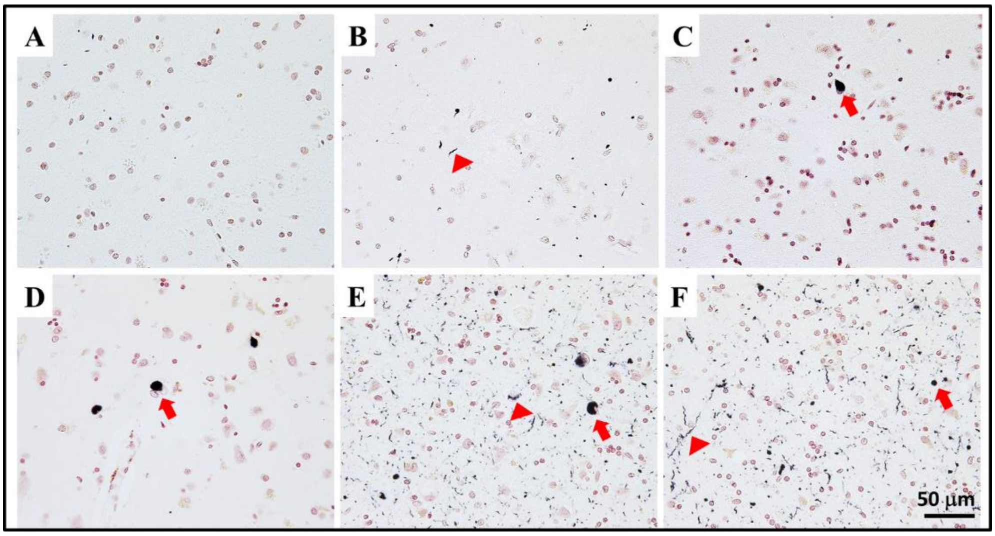

Figure S3. Representative images of p-αSyn-immunoreactive Lewy body (LB) and Lewy neurites (LN) in neutral red counter-stained temporal cortex. From A–F demonstrate LB and LN density from score 0–4. Score 4 images were shown in E,F from two fields from the same case to show the abundance of both LB and LN. Representative LB profiles are indicated by arrows and LN by arrowhead. Calibration bar is shown in F.
